# Draft Genome Sequences of 9 Actinobacteria from the Family *Microbacteriaceae* Associated with Insect- and Nematode-Damaged Plants

**DOI:** 10.1128/mra.00487-22

**Published:** 2022-08-31

**Authors:** Sergey V. Tarlachkov, Yury V. Ospennikov, Alexander V. Demidov, Irina P. Starodumova, Lubov V. Dorofeeva, Natalia V. Prisyazhnaya, Vladimir N. Chizhov, Sergei A. Subbotin, Lyudmila I. Evtushenko

**Affiliations:** a All-Russian Collection of Microorganisms (VKM), G.K. Skryabin Institute of Biochemistry and Physiology of Microorganisms, Pushchino Scientific Center for Biological Research, Russian Academy of Sciences, Pushchino, Russia; b Center of Parasitology of A.N. Severtsov Institute of Ecology and Evolution, Russian Academy of Sciences, Moscow, Russia; c California Department of Food and Agriculture, Sacramento, California, USA; University of Arizona

## Abstract

Draft genome sequences of 9 strains of known and putative new species of *Microbacteriaceae* isolated from insect- and nematode-damaged plants were generated using Illumina technology. The data obtained will contribute to the development of the genome-based prokaryote taxonomy and the knowledge on the biology of the microbial group investigated.

## ANNOUNCEMENT

Actinobacteria of the family *Microbacteriaceae* are distributed widely in various terrestrial and aquatic environments and often inhabit plants as endophytes or pathogens (1–3). Many putative new species of several genera of this family, along with representatives of known species, were found in plants infested by different nematodes (4, 5).

Novel strains were isolated from affected and unaffected parts of leaves of different herbaceous and woody plants infected by nematodes, leaf-mining insects, and plant-parasitic mites (Table 1).

**TABLE 1:** Strain data, characteristics, and DDBJ/ENA/GenBank accession numbers of genome sequences

Organism	Plant	Plant parasite	Geography	No. of reads	Coverage (×)	No. of scaffolds	Scaffold *N*_50_ (bp)	Genome size (Mbp)	G+C content (%)	No. of proteins	Closest species by 16S rRNA gene (identity %)	Closest species by ANI (identity %)	Closest species by dDDH (identity %)	16S rRNA gene GenBank accession no.	Genome GenBank accession no.	Genome SRA accession no.
Clavibacter michiganensis VKM Ac-2921	*Tilia* sp.	Eriophyes tiliae	Moscow region, Russia	14,417,302	660	8	2,063,997	3.18	73.4	2,996	Clavibacter michiganensis subsp. phaseoli (100)	Clavibacter michiganensis (97.30)	Clavibacter michiganensis (77.2)	ON158671.1	JALGRN000000000	SRR18694026
*Curtobacterium* sp. VKM Ac-2922	Fern	*Aphelenchoides* sp.	Washington state, USA	13,428,696	526	29	222,655	3.73	71.0	3,501	Curtobacterium flaccumfaciens (99.93)	Curtobacterium flaccumfaciens (82.16)	Curtobacterium flaccumfaciens (25.4)	ON158672.1	JALGRO000000000	SRR18694025
*Microbacterium* sp. VKM Ac-2923	Tellima grandiflora	*Aphelenchoides* sp.	Munster, Germany	14,406,260	536	37	362,450	3.93	69.8	3,531	Microbacterium testaceum (100)	Microbacterium proteolyticum (87.05)	Microbacterium proteolyticum (33.0)	ON158673.1	JALGRP000000000	SRR18694024
Rathayibacter caricis VKM Ac-2924	*Umbelliferae* ^*a*^	Unknown insect (leaf miner)	Moscow region, Russia	12 318 900	426	23	880,020	4.21	71.5	3,925	Rathayibacter caricis (99.79)	Rathayibacter caricis (97.71)	Rathayibacter caricis (82.9)	ON158674.1	JALGRQ000000000	SRR18694023
Rathayibacter festucae VKM Ac-2925	*Umbelliferae* ^*a*^	Unknown insect (leaf miner)	Moscow region, Russia	14,839,660	479	7	2,978,450	4.49	72.2	4,073	Rathayibacter festucae (100)	Rathayibacter festucae (97.80)	Rathayibacter festucae (80.1)	ON158675.1	JALGRR000000000	SRR18694022
*Rathayibacter* sp. VKM Ac-2926	*Umbelliferae* ^*a*^	Unknown insect (leaf miner)	Moscow region, Russia	17,536,744	590	30	366,154	4.32	72.3	3,873	Rathayibacter festucae (100)	Rathayibacter festucae (96.17)	Rathayibacter festucae (67.8)	ON158676.1	JALGRS000000000	SRR18694021
*Rathayibacter* sp.VKM Ac-2927	*Tilia* sp.	Unknown insect (leaf miner)	Moscow region, Russia	18,677,542	598	13	748,896	4.55	72.1	4,169	Rathayibacter festucae (100)	Rathayibacter festucae (96.18)	Rathayibacter festucae (67.9)	ON158677.1	JALGRT000000000	SRR18694020
*Rathayibacter* sp. VKM Ac-2928	*Aesculus* sp.	Cameraria ohridella (leaf miner)	Moscow region, Russia	13,232,092	446	13	849,744	4.32	72.3	3,887	Rathayibacter festucae (100)	Rathayibacter festucae (96.17)	Rathayibacter festucae (67.8)	ON158678.1	JALGRU000000000	SRR18694019
*Rathayibacter* sp. VKM Ac-2929	*Artemisia* sp.	Unknown insect (leaf miner)	Moscow region, Russia	16,522,532	529	19	764,283	4.53	72.1	4,129	Rathayibacter festucae (100)	Rathayibacter festucae (96.03)	Rathayibacter festucae (67.7)	ON158679.1	JALGRV000000000	SRR18694018

a Unaffected parts of leaves were used for the isolation of strains.

The air-dried plant samples were soaked in distilled water for 1 h, washed twice with sterile distilled water, placed in 0.85% NaCl solution, and milled. One drop of the obtained suspension was plated onto Reasoner’s 2A (R2A) agar (Fluka Analytical, USA) and incubated for 1 to 3 weeks at room temperature (18 to 24°C). The universal bacterial primers 27F (5′-AGAGTTTGATCCTGGCTCAG-3′) and 1525R (5′-AAGGAGGTGATCCAGCC-3′) with standard cycling conditions (95°C for 5 min; 30 cycles at 94°C for 30 s, 55°C for 30 s, and 72°C for 1 min 20 s; and 72°C for 4 min) were used for 16S rRNA gene PCR amplification followed by sequencing. Preliminary identification based on nearly entire 16S rRNA gene sequences revealed that the newly isolated strains belong to four genera of the family *Microbacteriaceae*, exhibiting high sequence identities to the known species (Table 1).

For genome sequencing, biomass was grown in liquid peptone-yeast-glucose (PYG) medium (5 g peptone, 3 g yeast extract, 5 g glucose, 0.2 g KH_2_PO_4_, and 1 L distilled water [рН 7.2]) or on R2A agar inoculated with cells from a single colony, followed by cultivation at 28°C for 2 to 3 days. Genomic DNA was extracted using a QIAamp DNA minikit (Qiagen, Germany). DNA library construction and sequencing were performed by using Novogene Co., Ltd. Libraries were generated with the NEBNext DNA library prep kit for Illumina (New England Biolabs, USA) following the manufacturer’s recommendations. Pooled DNA libraries were sequenced on an Illumina NovaSeq 6000 instrument to obtain 150-bp paired-end reads.

The quality of the reads was checked with FastQC v0.11.8 (https://www.bioinformatics.babraham.ac.uk/projects/fastqc/). Adapter sequences and low-quality regions in raw reads were cut with Trimmomatic 0.39 (6). Trimmed reads were assembled using SPAdes 3.15.4 (7). Assemblies were annotated with NCBI PGAP 6.1 (8). The pairwise similarity between the 16S rRNA gene sequences was determined using TaxonDC 1.3.1 (9). The genome relatedness indices, *viz.*, the average nucleotide identity (ANI) and digital DNA-DNA hybridization (dDDH) values, were calculated using JSpecies 1.2.1 (10) and GGDC 2.1 (11) tools, respectively. Accession numbers and characteristics of genomes are provided in Table 1.

The ANI and dDDH values obtained for strains VKM Ac-2922 and VKM Ac-2923 and their closest relatives were well below the accepted species threshold (12), indicating the strains to be members of two putative new species in the genera *Curtobacterium* and *Microbacterium.* Four strains (VKM Ac-2926, VKM Ac-2927, VKM Ac-2928, and VKM Ac-2929) may represent a new species as well. The genome relatedness indices for these strains toward their closest relative, namely, Rathayibacter festucae, were at the borderline for species definition or slightly below (12). From the genome relatedness indices, three remaining strains (VKM Ac-2921, VKM Ac-2924, and VKM Ac-2925) belonged to the known *Clavibacter* and *Rathayibacter* species.

### Data availability.

The whole-genome shotgun projects and the 16S rRNA gene sequences have been deposited in DDBJ/ENA/GenBank under the accession numbers listed in Table 1.
